# Unusual presentation; seeding of tumor after biopsy for hepatocellular carcinoma

**DOI:** 10.12669/pjms.326.11291

**Published:** 2016

**Authors:** Asude Aksoy, Koray Karabulut, Hakan Artas, Ahmet Kilicarslan, Sertac Usta, Ibrahim Halil Bahcecioglu

**Affiliations:** 1 Asude Aksoy, Medical Oncology Division, Firat University, Medical Faculty, Elazig, Turkey; 2 Koray Karabulut, Division of General Surgery, Firat University, Medical Faculty, Elazig, Turkey; 3 Hakan Artas, Division of Radiology, Firat University, Medical Faculty, Elazig, Turkey; 4 Ahmet Kilicarslan, Harput State Hospital, Division of Pathology, Elazig, Turkey; 5 Sertac Usta, Division of General Surgery, Firat University, Medical Faculty, Elazig, Turkey; 6 Ibrahim Halil Bahcecioglu, Division of Gastroenterology, Firat University, Medical Faculty, Elazig, Turkey

**Keywords:** Biopsy, Hepatocellular carcinoma, Seeding

## Abstract

Hepatocellular cancer is one of the most common and fatal cancer tumor worldwide. However, the obtained results are questionable in terms of medical treatment of hepatocellular cancer. The muscle, soft tissue and cutaneous metastases of hepatocellular cancer, for instance, are rare and may result from interventional procedures. Seeding of tumor along the biopsy needle upon percutaneous biopsy is a very rare phenomenon. We report a very rare case of a 79 -year- old man, known to be hepatitis C virus carrier with a metastatic tumor in abdominal wall caused by seeding of tumor after three years following a percutaneous biopsy procedure. Even years later, after a biopsy procedure for diagnostic purposes and may be soft tissue metastases. This complication is a very rare condition that should not be ignored but can be observed. The biopsy requirement should be questioned closely and avoided unnecessary biopsy procedures.

## INTRODUCTION

Hepatocellular cancer is the 6^th^ most common solid tumor, and the 3^rd^ most common fatal tumor worldwide.^1^ Based on the European Association for the Study of Liver Diseases (EASL) and American Association for the Study of Liver Diseases (AASLD) guidelines, nodules with a diameter >2 cm and typical vascular pattern can be diagnosed by computed tomography (CT) or magnetic resonance imaging (MRI) without biopsy. For therapeutic decision making, HCC diagnosis should be confirmed by biopsy for lesions with a diameter of 1 to 2 cm and typical vascular pattern detected by contrast CT or MRI, or atypical appearance.^2^-^4^ Fine needle aspiration is associated with a smaller risk of tumor invasion compared to biopsy.^5^ We report a case of abdominal wall seeding that occurred inadequate biopsy after three years for the diagnosis of HCC.

## CASE REPORT

In February 2011, a 79-year-old male patient with no history of alcohol use visited a private hospital with a complaint of abdominal pain, and biopsy was performed twice for the mass of 2x3x4 cm located in left lobe of liver. Both biopsies were considered inadequate. It was found to be positive for anti-HCV, he had normal results for liver function tests and tumor marker investigations, and he was observed without any medical treatment. He had no complaint in follow-up.

**Fig.1:**
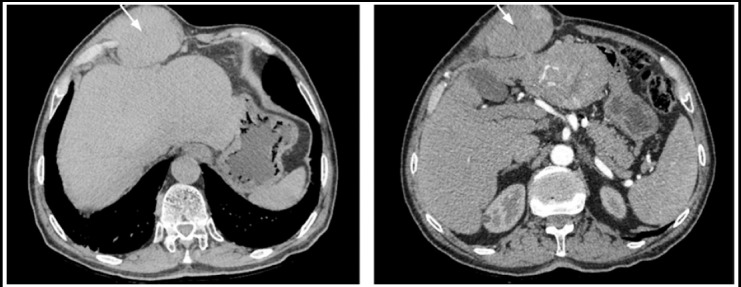
Abdominal computed tomography, hypervascular, solid, non-homogenous mass of 10 X 7 X 12 cm size adjacent to thoracic abdominal wall in hepatic left lobe, segment 4.

**Fig.2:**
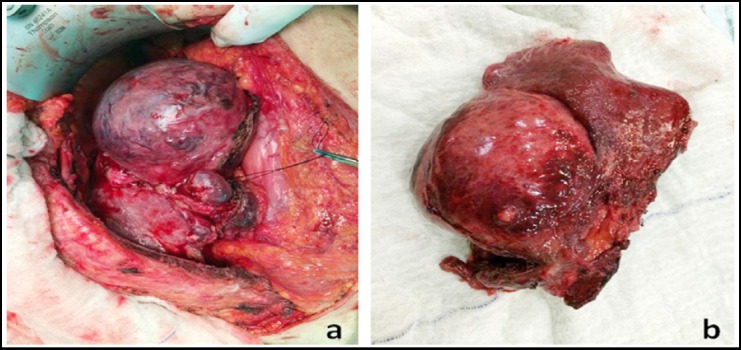
The material of hepatic resection, macroscopically.

**Fig.3:**
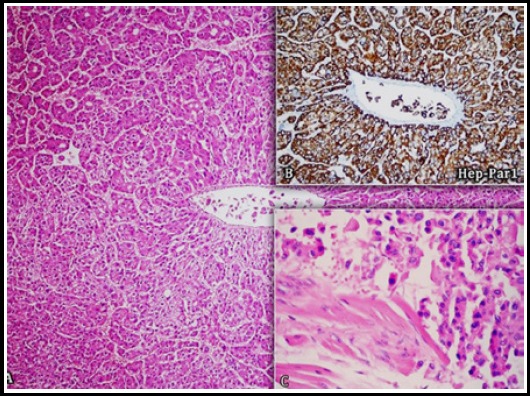
Histopathological examination. Hepatocellular carcinoma exhibiting moderate differentiation. A.) Cord structures and tumor cells of intense eosinophilic cytoplasm and round nuclei with an acinar pattern (upper left) in some regions. B.) Granular HEPPAR1 immunoreactivity in the cytoplasm of the tumor cells. C.) Striated muscle up take. (H&E, X 200; immunoperoxsidase, X 400; C: H&E, X 400).

In 2014, the patient came to our hospital with a palpable, painless mass in the right upper quadrant; liver function tests and level of alpha fetoprotein were normal, and abdominal tomography showed a highly vascularized mass of 10x7x12 cm with irregular contours located in thoracic-abdominal wall at the left lobe of liver, segment 4, with multiple lesions of 2 cm in size adjacent to this lesion in the intra-abdominal and sub-hepatic regions on the right side (Fig.1). It was thought that the mass in rectus muscle could be advanced depending on seeding after old biopsies and subsequently, surgery was planned. The patient underwent left hepatectomy, resection of abdominal wall and reconstructioned of abdominal wall by using dual mesh. The hepatic partial resection material was deemed as hepatocellular carcinoma (Fig. 2a, 2b). The mass in the anterior abdominal wall represented hepatocellular carcinoma seeding of tumor and omentum biopsy revealed that it was hepatocellular carcinoma metastasis. The pathological stage was T4; hepatic capsule was intact; and angio-lymphatic and per neural invasion were present. The result was reported as two tumor nodules in the liver, the larger one of 9.2 cm in size and the smaller one of 0.4 cm in size with tumor seeding of 11.5 cm in the anterior abdominal wall, and a clean surgical border (Fig.3). The patient was followed for approximately two years without recurrence.

## DISCUSSION

The patient known to be a Hepatitis C carrier exhibited no clear results upon inadequate biopsy sampling performed three years ago, had normal results in liver function tests, and he was observed without any medical treatment. For the last three months, patient who had right flank pain and the mass on the right side, levels of AFP and liver function tests the patient’ s were normal marked. Biopsy was performed to explain the etiology of the patient’s right hand side of the painless mass. We realized that the tumoral seeding had occurred due to inconclusive biopsy sampling performed three years ago according to pathologist’s report. Seeding of tumor after interventional procedure is rather rare.

Seeding of tumor may occur at a rate of 2.29% biopsy with percutaneous ethanol injection, 1.4% alone liver biopsy, 0.95% radiofrequency ablation with biopsy, and 0.61% radiofrequency ablation without biopsy.^6^ However, in the other study reported that this incidence was 0.14%.^7^ Studies have shown that seeding of tumor, following interventional procedures, may occur in the presence of risk factors including sub capsular localization of the tumor nodule, a low degree of differentiation, a high alpha fetoprotein level and a tumor diameter > 5 cm.^8^

Another trial has also shown that the risk of seeding within 3 months and 4 years following biopsy was between 0% and 5.1%. In the report of studies that the patients should be monitored for a minimum of 4 years for the presence of such a risk.^5^ In our patient, the diagnosis of HCC could be confirmed 3 years after the biopsy procedure; and the patient’s mass in left hepatic lobe anterior section was initially of 2x3x4 cm in size, and he had undergone two unsuccessful biopsies. The use of large-diameter needles for radiofrequency ablation and the lack of using coaxial cutting techniques may increase the risk for seeding of tumor following the procedure.

The most of time radiological imaging may be sufficient for diagnosis of HCC and biopsy procedure shouldn’t be administered except in very exceptional circumstances. If a biopsy is performed, it would be remembered that there is a risk of seeding and this situation may occur years even after the procedure. If the biopsy is done, the procedure should be performed by qualified professionals, by using appropriate technique and needles in unique expedition. While seeding of tumor following biopsy is an extremely rare phenomenon, it should not be overlooked.
